# Rhabdomyolysis-induced acute kidney injury from a small number of wasp stings

**DOI:** 10.1590/0037-8682-0313-2022

**Published:** 2023-01-23

**Authors:** Kenan Fang, Jingwen Ni, Junyu Dong

**Affiliations:** 1 Pediatric intensive care unit, Luoyang Maternal and Child Health Hospital, Luoyang, China.

**FIGURE 1:**
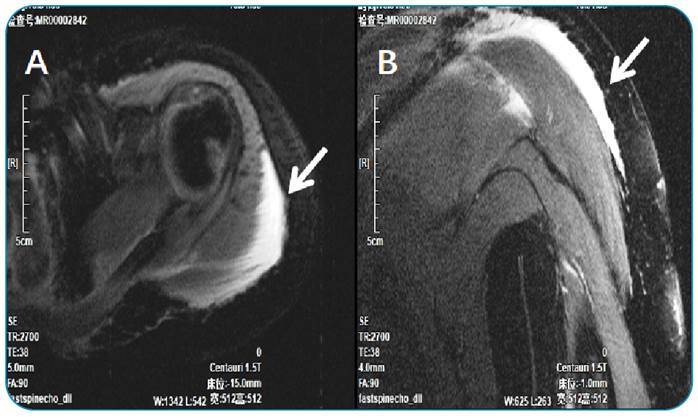
**(A, B):** MRI scans reveal swelling in the deltoid, trapezius, and supraspinatus muscles and subcutaneous fat layer (arrow).

An 11-year-old boy was admitted to the hospital after being stung 11 times by wasps. Physical examination revealed lethargy, irritability, puffy eyelids, swollen legs, and necrotic ulcers around the sting sites. Laboratory tests showed the following: white blood cell, 35.18×10^9^/L; neutrophil, 90%; creatine kinase (CK), 2546.2 U/L; lactate dehydrogenase, 3009.5 U/L; myoglobin (Mb), 1935 ng/ml; alanine transaminase, 1016 U/L; aspartate transaminase, 3225.4 U/L; activated partial thromboplastin time, 78.5 s; protein 3+; occult blood 3+; urobilinogen 1+; and bilirubin 3+. The patient received epinephrine, furosemide, and fluid replacement therapy. A build-up of fluid became evident and the patient was transferred to continuous blood purification therapy owing to the low urine output (0.4 mL/kg/h)^1^ ^-^ ^3^. Urine color changed from brown to yellow 24 h later, the patient regained consciousness 48 h later, and urine output reached 1 mL/kg/h 56 h later. The serum levels of CK and Mb reached maximum values after 96 h and fell within the normal ranges after 1 week. In the ultrasound images, the striated muscle texture was blurry and showed ground-glass opacity. Magnetic resonance imaging revealed muscle swelling Figure 1 (A, B). The severity of the reaction to a wasp-sting is closely associated with the number of stings. More than 50 stings may lead to acute kidney injury (AKI), rhabdomyolysis, hemolysis, shock, and even death. This case shows that a few wasp stings can cause rhabdomyolysis and AKI and that close attention should be paid to the rate of change in CK and Mb levels after a sting.
